# Effects of toxic cellular stresses and divalent cations on the human P2X7 cell death receptor

**Published:** 2008-05-19

**Authors:** Mélody Dutot, Hong Liang, Thierry Pauloin, Françoise Brignole-Baudouin, Christophe Baudouin, Jean-Michel Warnet, Patrice Rat

**Affiliations:** 1 Laboratoire de Toxicologie, Faculté des Sciences Pharmaceutiques et Biologiques, Université Paris Descartes, Paris, France; 2 INSERM UMRS 872, Institut Biomédical des Cordeliers, Paris, France; 3 Centre Hospitalier National d’Ophtalmologie des Quinze-Vingts, Paris, France; 4 Université de Versailles, Versailles, France

## Abstract

**Purpose:**

The purpose of this study was to investigate responses to toxic cellular stresses in different human ocular epithelia.

**Methods:**

Reactivity with a specific anti-P2X7 antibody was studied using confocal fluorescence microscopy on conjunctival, corneal, lens, and retinal cell lines as well as using impression cytology on human ocular cells. Activation of the P2X7 receptor by selective agonists (ATP and benzoylbenzoyl-ATP) and inhibition by antagonists (oATP, KN-62, and PPADS) were evaluated using the quinolinium,4-[(3-methyl-2-(3H)-benzoxazolylidene) methyl]-1-[3-(triethylammonio)propyl]di-iodide (YO-PRO-1) test in cytofluorometry. Different specific stresses were then induced by a chemical toxin (benzalkonium chloride) and a chemical oxidant (tert-butyl hydroperoxide) to assess the role of the P2X7 receptor. Modulation of P2X7 receptor activation was performed with several ionic solutions.

**Results:**

Our data show that four cell lines express the P2X7 cell death purinergic receptor as judged by reactivity with a specific anti-P2X7 antibody, activation by the selective P2X7 agonist benzoylbenzoyl-ATP and to a lesser extent by ATP (YO-PRO-1 dye uptake), and inhibition by three antagonists (oATP, KN-62, and PPADS). Benzalkonium chloride, a widely used preservative, induced dramatic membrane permeabilization through P2X7 pore opening on conjunctival and corneal epithelia. Reactive oxygen species, induced by tert-butyl hydroperoxide, lead to P2X7 receptor activation on retinal pigment epithelium. Modulation of P2X7 receptor activation was obtained with extracellular Ca^2+^ and Mg^2+^ and with a controlled ionization marine solution rich in different divalent cations. This marine solution could be proposed as a new ophthalmic solution.

**Conclusions:**

Our observations reveal a novel pathway for epithelial cells apoptosis/cytolysis by inducing different toxic stresses and their modulation by using ionic solutions.

## Introduction

P2X receptors are ligand-gated ion channels that are activated by extracellular ATP. Their activation results in the opening of a cationic channel with significant permeability to calcium and intracellular depolarization [1,2]. P2X receptors have two transmembrane domains with short intracellular NH_2_- and COOH-termini. The last member of this family, the P2X7 receptor, differs from the other P2X receptors in a distal COOH-terminal region. Truncations in this region result in non-functional receptors with no cell surface expression [3].

Exposure to ATP or to the more potent agonist, 2’-3′-O-(4-benzoyl)benzoyl-ATP (BzATP), renders the P2X7 receptor permeant to ions, and repeated or prolonged application of either agonists induces the formation of a cytolytic pore that is permeable to larger molecules (up to 900 Da) such as fluorescent dyes quinolinium,4-[(3-methyl-2-(3H)-benzoxazolylidene) methyl]-1-[3-(triethylammonio)propyl]di-iodide (YO-PRO-1) within a few seconds [4,5]. One of the characteristic features of the P2X7 receptor is its inhibition by extracellular divalent cations [2,4,6,7].

The P2X7 receptor is expressed in very different tissues, and its activation can trigger multiple cellular responses. The P2X7 receptor is implicated in inflammation through the induction of pro-inflammatory cytokine release (mainly interleukin-1 and interleukin-6) [8,9]. Furthermore, the P2X7 receptor can play a key role in apoptosis and cytolysis through the activation of caspases, p38 mitogen-activated protein (MAP) kinase, extracellular signal-regulated kinases (ERKs), and c-Jun kinase [10-12].

The eye is a very sensitive organ that is the site of a wide range of disorders. Not only can the active principle of medications be responsible for eye irritation but also the excipients [13]. For example, the toxicity of the benzalkonium chloride (BAC) preservative, a quaternary ammonium, has been widely documented [14-16]. Long-term treatment with preserved eye drops can lead to the deepithelialization of the ocular surface [17].

The retinal pigment epithelium monolayer is at risk for oxidative damage due to its location in a highly oxygenated environment and its exposure to high levels of visible light. Although visible light does not damage cells by directly interacting with DNA and most proteins, it can lead to oxidation of key constituents via reactions with endogenous photosensitizers. Retinal epithelium is therefore likely to accumulate oxidative damage over time, which is believed to cause tissue dysfunction that may contribute to diseases of aging.

Ocular epithelia can then be damaged by a wide range of exogenous chemical and physical toxic agents, but the induced cell mechanisms remain unknown.

Gröschel-Stewart et al. [18] identified the P2X7 receptor in numerous epithelia in the rat including the cornea, esophagus, soft palate, tongue, vagina, and foot pad, concluding that P2X7 receptor could represent a target for the development of therapeutics in the treatment of epithelial dysfunctions. To our knowledge, no study has been run on human epithelia. Our aim was to study the P2X7 receptor in four human ocular epithelia to evaluate its implication in different toxicological pathologies. The potency of several ionic solutions to modulate P2X7 receptor activation was also assessed.

## Methods

### Reagents

Materials for cell culture were provided by Eurobio (Les Ulis, France). ATP, BzATP, oATP (2',3′,-dialdehyde ATP), PPADS (pyridoxal-phosphate-6-azophenyl-2′,4′-disulphonic acid), KN-62 (1-(N,O-bis[5-isoquinolinesulphonyl]-N-methyl-L-tyrosyl)-4-phenylpiperazine), benzalkonium chloride, tert-butyl hydroperoxide, CaCl_2_, MgCl_2_, anti-P2X7 receptor antibody, mouse anti-rabbit IgG-FITC conjugate, ATP bioluminescent assay kit, and neutral red dye were from Sigma-Aldrich (Saint-Quentin Fallavier, France). Alexa Fluor® 488 goat anti-rabbit IgG, propidium iodide, YO-PRO-1, and 2’,7’-dichlorofluorescein diacetate were purchased from Invitrogen (Carlsbad, CA). Brilliant Blue G (BBG) was purchased from Bio-Rad (Richmond, CA).

The controlled ionization marine solution (Lacrymer®) was purchased from Yslab (Quimper, France).

### Cell culture

A human conjunctival cell line (WKD, ECACC 93120839) was cultured under standard conditions (moist atmosphere of 5% CO_2_ at 37 °C) in Dulbecco’s minimum essential medium (DMEM) supplemented with 10% fetal bovine serum (FBS), 2 mM L-glutamine, 50 IU/ml penicillin, and 50 IU/ml streptomycin. The medium was changed every three days. Confluent cultures were removed by trypsin incubation, and then cells were counted. They were seeded into 96-well culture microplates at a density of 90,000 cells per well for microtitration analysis. Cultures were kept at 37 °C for 24 h.

A human corneal cell line (HCE, RCB 1384) [19] was cultured under standard conditions in a mixture 1:1 of DMEM and Nutrient Mixture F12 supplemented with 10% FBS, 2 mM L-glutamine, 50 IU/ml penicillin, and 50 IU/ml streptomycin. The medium was changed every two days. Confluent cultures were removed by trypsin incubation, and then cells were counted. They were seeded into 96 well culture microplates at a density of 70,000 cells per well and were kept at 37 °C for 24 h.

A human retinal pigmented epithelium cell line (ARPE-19, ATCC CRL2302) was cultured in a mixture 1:1 of DMEM and Nutrient Mixture F12 supplemented with 10% FBS, 2 mM L-glutamine, 50 IU/ml penicillin, and 50 IU/ml streptomycin. The medium was changed every two days. Confluent cultures were removed by trypsin incubation, and then cells were counted. They were seeded into 96 well culture microplates at a density of 20,000 cells/ml and kept at 37 °C for 24 h.

A human lens epithelial (HLE) cell line, SRA 01/04, obtained from Washington University, (St Louis, MO) was cultured under standard conditions in 1% glucose DMEM supplemented with 15% FBS, 2 mM glutamine, and 4 µg/ml gentamicin. They were seeded into 96 well culture microplates at a density of 30,000 cells per well and were kept at 37 °C for 24 h.

### Impression cytology (ex vivo human conjunctival epithelium)

Informed consent that was approved by the Institutional Review Board was obtained from each normal volunteer, and research was performed according to the tenets of the Declaration of Helsinki. Criteria for participation included persons of either gender, at the age of 18 years or older, and with no known external ocular disorders.

To obtain specimens, a paper filter (Gelman Supor®, Pall Sciences, Saint-Germain-en-Laye, France) was applied on the conjunctiva to collect the most superficial layers of the ocular surface. The paper filter was then allowed to dry completely for at least 24 h.

### P2X7 receptor localization by confocal microscopy

Cell lines were cultured on slides (Laboratory-tek II chambered coverglass; Nalge Nunc International, Naperville, IL). Culture cells and impression cytology specimens were fixed with 4% paraformaldehyde and permeabilized with Triton X-100. Cells were immersed for 30 min in 1% BSA followed by a 1 h incubation time with the rabbit anti-P2X7 receptor antibody. Cells were then incubated with a secondary antibody, Alexa Fluor® 488 goat anti-rabbit IgG, for 1 h at room temperature. Propidium iodide was added to mark cell nuclei before examination with a confocal epifluorescence microscope (E800, PCM 2000; Nikon, Tokyo, Japan).

### ATP release evaluation by luminescence

Quantitative determination of ATP was assessed using the ATP bioluminescent assay kit from Sigma-Aldrich. Briefly, the cells were incubated with BAC for 15 min, and supernatants were collected for ATP quantification. Luminescence was measured using a spectral scanning reader (Varioskan Flash, Thermo Electron Corporation, Saint-Herblain, France). 

### P2X7 receptor activity evaluation by microcytofluorometry

Experiments were conducted using microplate fluorometry, which allows fluorometric detection (230–1000 nm) with high sensitivity (pg-fg/ml; Safire™; Tecan, Lyon, France). This technique allows the use of fluorescent probes directly on living cells and detects the fluorescent signal directly in the microplate in less than 1 min (for a 96 well microplate).

YO-PRO-1, a DNA probe, only enters apoptotic cells after P2X7 receptor activation-induced pore formation. A 2-μM YO-PRO-1 solution in phosphate buffer saline (PBS) was distributed in the wells (200 μl per well), and the microplate was placed at room temperature in the dark. After 10 min, the fluorescence signal was scanned (excitation [ex]=491 nm; emission [em]=509 nm). For kinetic studies, YO-PRO-1 dye was directly diluted in the xenobiotic solutions.

### Cell viability and oxidative stress evaluation by microcytofluorometry

Membrane integrity, which is closely correlated with cell viability, was evaluated with neutral red (NR) using fluorometric detection (ex=535 nm; em=600 nm).

Reactive oxygen species (ROS) were detected with the 2’,7’-dichlorofluorescein diacetate probe (DCFH-DA). The fluorescent signal (ex=485 nm; em=535 nm) is proportional to ROS production.

### Data analysis

Data in the text and graphs are shown as relative fluorescence units or as fluorescence percentage of the control, and statistical analyses were performed using one-way ANOVA followed by Dunnett’s test (α risk=0.05). Each test was performed in triplicate.

## Results

### P2X7 receptor expression and activation on ocular surface

**Figure 1:**
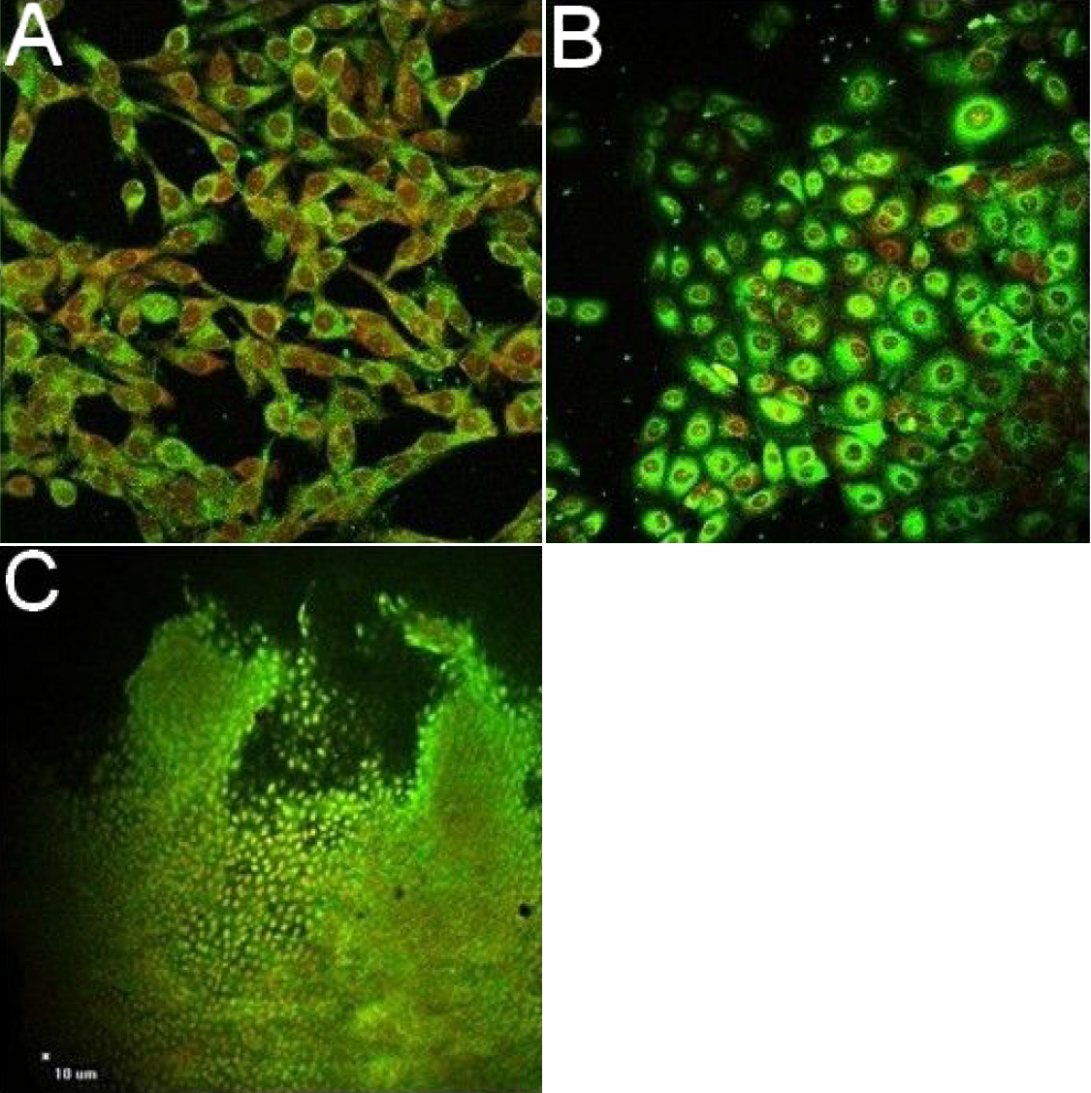
Expression of P2X7 receptor on human conjunctival and corneal epithelial cells. Conjunctival cell line (**A**), corneal cell line (**B**) and cells from ocular surface impression cytology (**C**) presented P2X7 receptor expression. Cells were fixed, permeabilized, and then incubated with a P2X7 antibody. Nuclei were stained with propidium iodide. Cells were observed using the confocal microscopy.

P2X7 expression was confirmed by confocal microscopy analysis. Figure 1A,B show strong labeling of conjunctival and corneal cell membrane using specific anti-P2X7 antibody that raised against the COOH-terminal epitope of the P2X7 receptor protein. Figure 1C shows immunoreactivity of human conjunctival cells from impression cytology. Isotype control monoclonal antibody was used to estimate the non-specific binding of target primary antibodies to cell surface antigens (data not shown).

**Figure 2:**
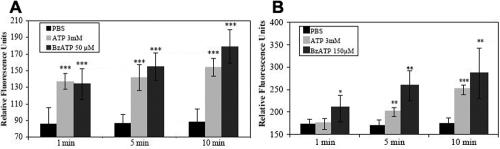
P2X7 receptor activation by ATP and BzATP. ATP and BzATP cause YO-PRO-1 dye uptake in conjunctival (**A**) and corneal cells (**B**). Cells were incubated with ATP or BzATP solutions containing YO-PRO-1.

Changes in conjunctival and corneal plasma membrane permeability due to stimulation with ATP and BzATP (which has a higher affinity for the P2X7 receptor) are shown in Figure 2A,B, respectively. As expected, BzATP induced a faster and more important permeabilization than ATP on both cell lines.

**Figure 3:**
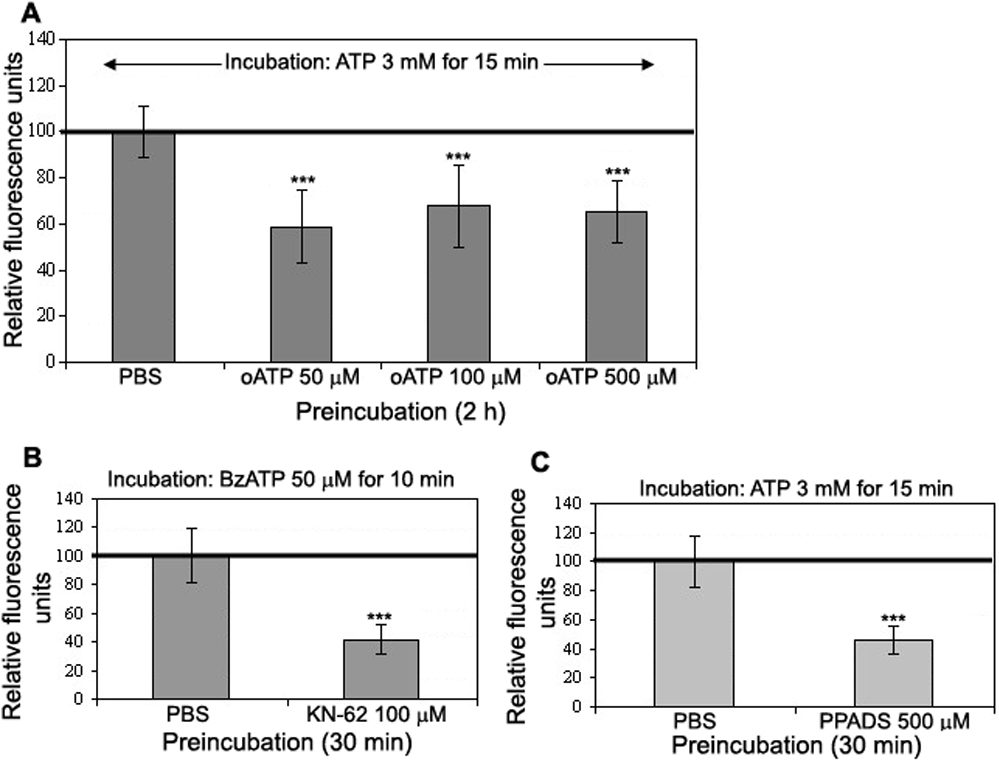
Results of YO-PRO-1 dye uptake test. oATP was pre-incubated for 2 h before ATP incubation and decreased YO-PRO-1 uptake (**A**); KN-62 was pre-incubated for 30 min before BzATP incubation and decreased YO-PRO-1 uptake (**B**); PPADS was pre-incubated 30 min before ATP incubation and decreased YO-PRO-1 uptake (**C**).

Different P2X7 receptor inhibitors were tested for their ability to block ATP or BzATP stimulation (Figure 3). Figure 3A shows that when pre-incubated for 2h, oATP (50 µM, 100 µM, and 500 µM) is able to significantly decrease YO-PRO-1 dye uptake after ATP stimulation by −41%, −33%, and 35%, respectively (no significant different between the three values). KN-62 (100 µM) and PPADS (500 µM) were pre-incubated for 30 min and show almost the same inhibition effect on P2X7 receptor stimulation (Figure 3B,C); they both induce an ~65% decrease in YO-PRO-1 uptake.

### P2X7 receptor implication in an iatrogenic pathology

**Figure 4:**
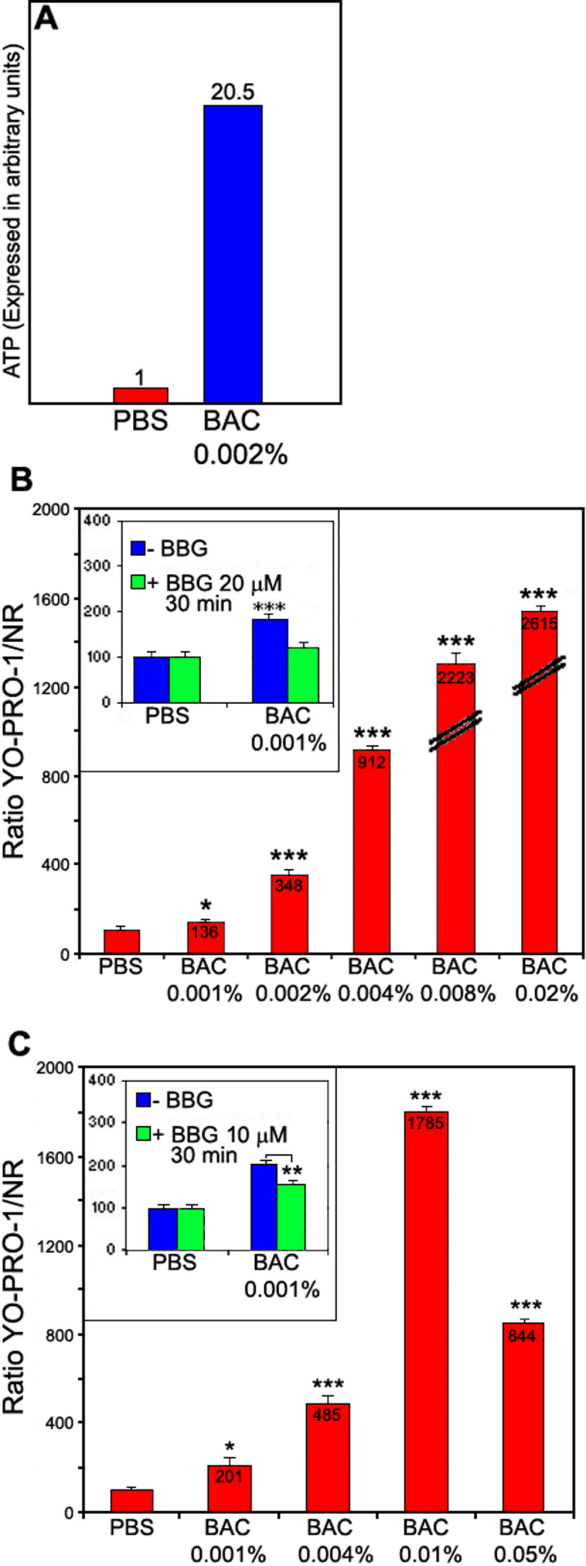
Effects of BAC on ocular surface cells. BAC induces ATP release in extracellular medium (**A**). The cells were incubated with 0.002% BAC for 15 min. Supernatants were collected for ATP quantification. BAC induces high YO-PRO-1 uptake in conjunctival (**B**) and corneal (**C**) cells. Frame: Cells were pre-incubated with 10 μM BBG for 30 min before 0.001% BAC and YO-PRO-1 incubation. BBG inhibited YO-PRO-1 uptake induced by BAC on both conjunctival (**A**) and corneal (**B**) cells. The asterisk indicates a p<0.05 compared to PBS and the triple asterisk denotes a p<0.001 compared to PBS.

ATP release after BAC incubation on conjunctival cells is shown in Figure 4A. After 15 min, BAC 0.002% induced a high ATP release in extracellular medium compared to PBS.

Figure 4A,B show BAC-induced permeabilization of the plasma membrane of conjunctival and corneal cells, respectively. YO-PRO-1/neutral red ratio was calculated to take into account the number of living cells responsible for YO-PRO-1 uptake. The lowest BAC concentration (0.001%) induced a P2X7 receptor activation leading to pore opening and YO-PRO-1 dye influx in both cell lines. When the cells were pre-incubated with 10 µM BBG (P2X7 receptor antagonist) before BAC, membrane permeabilization was inhibited on conjunctival cells (118%, not statistically different compared to PBS control) and on corneal cells (153% compared to PBS control versus 201% without BBG).

At 0.001% BAC, corneal cells seem to be more sensitive than conjunctival cells since BAC induced a higher increase in YO-PRO-1 fluorescence signal on the cornea than on the conjunctiva (201% versus 136%, respectively, compared to the control). At 0.004% BAC, conjunctival cells seem to be more sensitive than corneal cells (912% versus 485%, respectively, compared to the control). After incubation with 0.02% BAC on conjunctival cells and incubation with 0.05% BAC on corneal cells, the permeabilization reaches its maximum since YO-PRO-1 uptake does not increase compared to the lower BAC concentrations.

### P2X7 receptor expression and activation on retinal pigment epithelium

**Figure 5:**
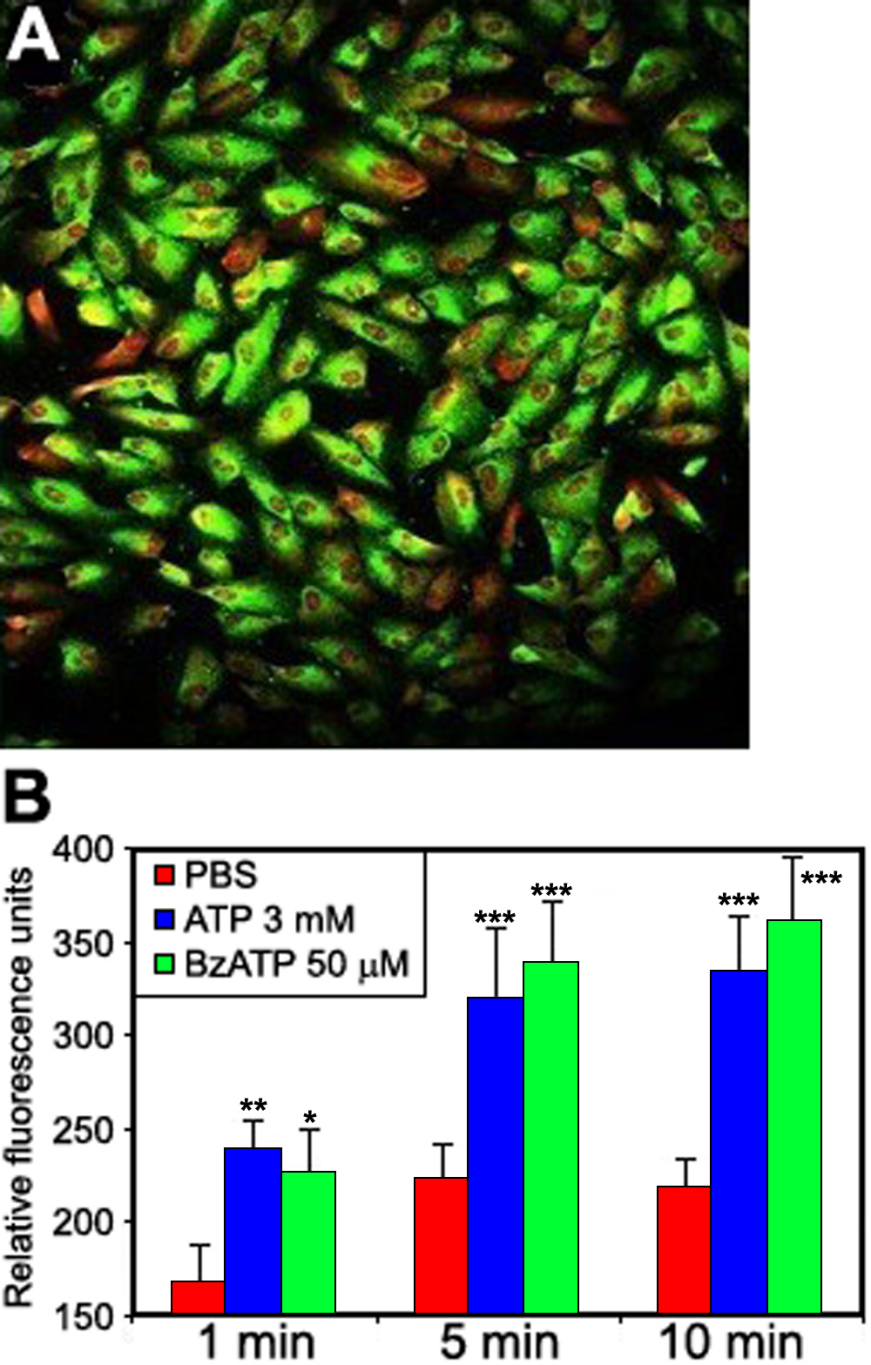
P2X7 receptor expression and activation on retinal epithelial cells. Cells were fixed, permeabilized, and then incubated with a P2X7 antibody (**A**). Nuclei were stained with propidium iodide. Cells were observed using the confocal microscopy. P2X7 receptor activation by ATP and BzATP causes YO-PRO-1 dye uptake in retinal cells (**B**).

Retinal pigment epithelial cells expressed the P2X7 receptor protein as shown in Figure 5A. Isotype control monoclonal antibody was used to estimate the non-specific binding of target primary antibodies to cell surface antigens (data not shown). ATP and BzATP stimulated P2X7 receptor activation (an increase in YO-PRO-1 dye uptake) with a more potent effect with BzATP than ATP (Figure 5B).

### P2X7 receptor implication in oxidative stress

**Figure 6:**
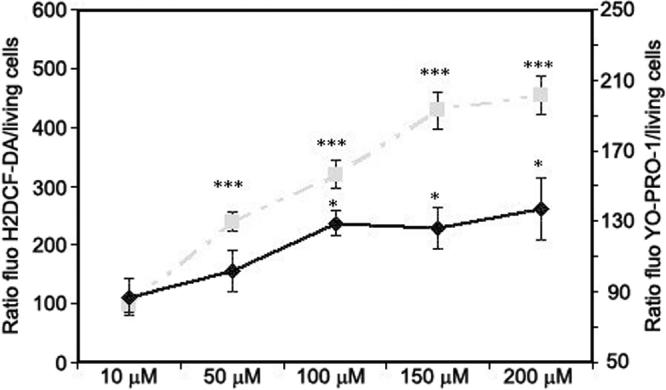
Effects of tBHP (chemical oxidant) on retinal epithelial cells. ROS overproduction (dashed line) and P2X7 receptor activation (full line) are induced by tBHP on retinal cells. Cells were exposed to various concentrations of tBHP for 1 h. ROS production and P2X7 pore formation were detected using the H2DCF-DA (dashed line) and YO-PRO-1 (full line) tests. The asterisk denotes a p<0.05 and the triple asterisk indicates a p<0.001 compared to PBS.

Tert-butyl hydroperoxide (tBHP) was chosen to induce oxidative stress on retinal pigment epithelial cells (Figure 6). tBHP (10 µM) induced neither ROS production nor P2X7 permeabilization. ROS overproduction appeared after 1 h incubation time with 50 µM tBHP whereas P2X7 permeabilization occurred after incubation with 100 µM tBHP.

### P2X7 receptor expression and activation on lens

**Figure 7:**
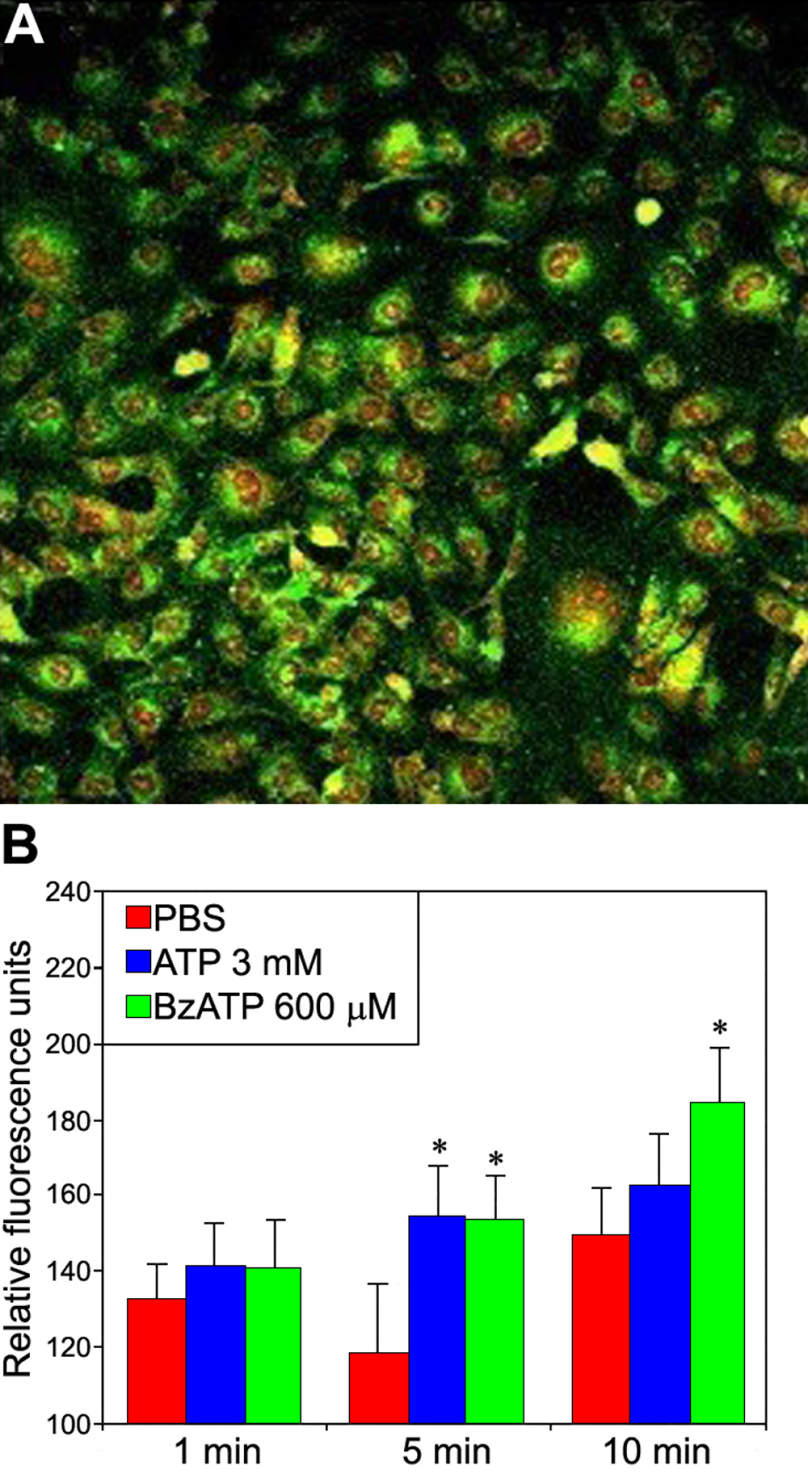
P2X7 receptor expression and activation on lens epithelial cells. Cells were fixed, permeabilized, and then incubated with a P2X7 antibody (**A**). Nuclei were stained with propidium iodide. Cells were observed using the confocal microscopy. P2X7 receptor activation by ATP and BzATP causes YO-PRO-1 dye uptake in lens cells (**B**).

P2X7 receptor expression and activation were also evaluated on lens cells. Figure 7A shows P2X7 protein localization using confocal microscopy (isotype control antibody was used to estimate the non-specific binding, data not shown), and Figure 7B confirms P2X7 receptor activity using the YO-PRO-1 uptake test after stimulation with ATP and BzATP. BzATP concentration (600 µM on lens cells) was 4–12 times higher than the concentrations used to stimulate corneal, conjunctival, and retinal cells.

### P2X7 receptor modulation with an ionic marine solution

**Figure 8:**
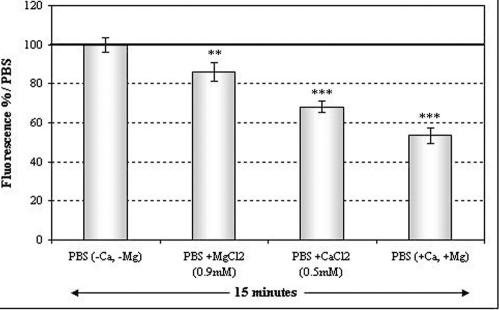
Effects of extracellular Ca^2+^ and Mg^2+^ on YO-PRO-1 uptake. Both Ca^2+^ and Mg^2+^ decreased YO-PRO-1 uptake but Ca^2+^ has a more potent effect than Mg^2+^. Ca^2+^ and Mg2+ together have a synergic effect on the decrease in YO-PRO-1 uptake. The double asterisk indicates a p<0.01 compared to PBS and the triple asterisk denotes a p<0.001 compared to PBS.

**Figure 9:**
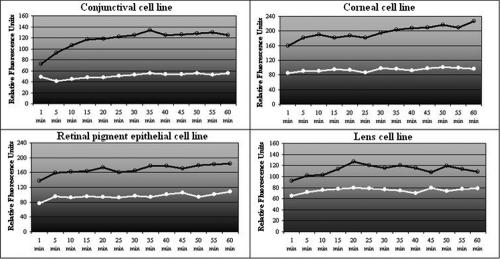
Effects of a controlled ionization marine solution on P2X7 receptor activation. Controlled ionization marine solution (CIMS) decreases YO-PRO-1 uptake in the four cell lines compared to PBS. The cells were incubated with either PBS (black line) or CIMS (white line).

As P2X7 receptor is a non-selective ion channel, the influence of divalent cations on P2X7 permeabilization was tested. PBS is commercially available with or without calcium and magnesium, so both types of PBS were incubated with cells. PBS containing calcium and magnesium (PBS +Ca, +Mg) induced less P2X7 basal permeabilization than PBS without calcium and magnesium (PBS -Ca, -Mg). MgCl_2_ and CaCl_2_ were dissolved in PBS -Ca, -Mg to the concentration corresponding to PBS +Ca, +Mg (0.5 mM and 0.9 mM, respectively). Figure 8 shows a synergic effect between Mg^2+^ and Ca^2+^, leading to a 50% decrease in the YO-PRO-1 fluorescence signal, whereas separately, Mg^2+^ and Ca^2+^ induced 14% and 32% decreases in the fluorescence signal, respectively. Since divalent cations seem to significantly modulate membrane permeabilization, the effect of a controlled ionization marine solution (derivative of sterile sea water) was studied (Figure 9). The controlled ionization marine solution decreased YO-PRO-1 uptake in the four cell lines compared to PBS.

## Discussion

The P2X7 receptor has been cloned and sequenced [2,20,21]. Its structure is well known, but the role of this receptor in ocular epithelia has not yet been studied yet. The expression of P2X7 receptor has so far been reported in immune cells, but little is known about its presence in epithelial cell types.

According to our results, P2X7 receptor is expressed in the four human epithelial cell lines we tested. Furthermore, we demonstrated the presence of P2X7 receptor in an original human ex vivo model, epithelial cells from impression cytology. The ability of ATP and BzATP to induce P2X7 pore opening was confirmed using 376 Da impermeant dye uptake (YO-PRO-1). Three different inhibitors were tested for their ability to block P2X7 receptor activation; they all decreased membrane permeabilization induced by ATP or BzATP. Most anti-P2X7 blockers are not absolutely specific to P2X7 receptors; rather they are P2X receptor inhibitors. Indeed, oATP is not specific to P2X7 since it also blocks currents at P2X1 and P2X2 receptors [22]. PPADS and KN-62 are much more specific to the human P2X7 receptor [23,24].

### P2X7 receptor is present and functional on human ocular epithelial cells

Conjunctival and corneal epithelia are very sensitive to external toxins far more than epidermal epithelium. At the moist surface of the body, the cornea is a non-keratinized stratified squamous epithelium contrary to keratin-rich epithelia. They can suffer many disorders since they are exposed to various external influences. Apart from physical stress, ocular medications (eye drops) are one of the most potent hazards of the ocular surface because they are in direct contact with the conjunctiva and the cornea. Our results showed that BAC, the most commonly used preservative in ophthalmology and in cosmetology, stimulated a loss of membrane integrity and then stimulated ATP release in extracellular medium. This ATP release could explain P2X7 receptor activation we observed through membrane permeabilization on both conjunctival and corneal cells. We could ask if the addition of an ATP-consuming enzyme (such as hexokinase) would inhibit BAC-induced YO-PRO-1 uptake. We confirm our previous work, which concluded that preserved fluoroquinolone eye drop intolerance was mainly due to P2X7 receptor activation, which was induced by BAC [25]. BBG, a P2X7 receptor antagonist, was able to block P2X7 activation induced by BAC. These results confirm the role of the P2X7 receptor in the cytotoxic mechanisms induced by BAC. We showed that as the BAC concentration increases, it becomes more hazardous for the conjunctiva. Cytolysis, induced by BAC [16], could be explained by a high activation of the P2X7 receptor, also known as the P2Z cytolytic receptor [1].

It seems that the P2X7 receptor mainly acts as a pro-apoptotic receptor in epithelia. Groschel-Stewart et al. [18] reported that the P2X7 receptor may play a role in the physiologic turnover of continuously regenerating epithelial tissue, and Greig et al. [26] observed that the P2X7 receptor could play a role in periderm cell apoptosis. Our work showing BAC-induced P2X7 receptor activation in the outer layers of the ocular surface epithelia are well correlated with these observations.

The optic nerve, which acts like a cable connecting the eye with the brain, is a continuation of the axons of the ganglion cells in the retina. As ATP can act as a neurotransmitter, high concentrations of P2X7 purinoreceptor in retinal cells are not surprising. The P2X7 receptor has been involved in neurodegenerative diseases [27]. The participation of reactive oxygen species in neurodegenerative diseases, mainly Alzheimer disease, is well documented [28]. Oxidative stress is also thought to be involved in retinal degeneration [29]. Therefore, we studied the role of P2X7 receptor in retinal cytotoxicity after oxidative stress induction. Indeed, neurodegenerative retinal diseases such as age-related macular degeneration (AMD) could be due to P2X7 receptor activation in the retina. Our results showed that an oxidant agent (tBHP) slightly induced P2X7 receptor activation. P2X7 activation was observed at higher doses of tBHP than those used to induce ROS overproduction. Therefore, we could imagine that ROS overproduction was responsible for P2X7 receptor activation. Effects of attacks by radicals, particularly those produced by ROS, can accumulate over the years [30], and ROS-induced P2X7 receptor activation could be part of cell death mechanisms that occur in neurodegenerative diseases.

The presence of an active P2X7 receptor in lens cells confirms the possible importance of this cytolytic receptor in different epithelial tissues.

Numerous reports suggest that the P2X7 receptor function can be regulated by extracellular ions [2,4,6,7]. Membrane permeability to fluorescent dyes was induced by ATP4- rather than MgATP2- [4]. It could then explain why divalent cations have the ability to inhibit pore formation by direct ionic interaction with the negative charges of ATP. In our study, Ca^2+^ was more effective in reducing P2X7 pore basal formation than Mg^2+^. This result is consistent with the atomic mass of calcium being higher than the magnesium atomic mass (40.078 against 24.305, respectively), which could explain why calcium induced a higher steric inhibition. A study on HEK293 cells stably expressing the rat P2X7 receptor showed that Mg^2+^ was more effective in inhibiting ATP-induced YO-PRO-1 uptake than Ca^2+^ [7] whereas a study on HEK293 cells expressing the human P2X7 receptor showed no significant difference between Mg^2+^ and Ca^2+^ in their capacity to inhibit BzATP-induced YO-PRO-1 uptake [6]. Our results, obtained with human cells, are not concordant with those observed with rat cells, but interspecies differences in the inhibiting effect of P2X7 receptor antagonists have already been reported [31]. Not only cations but also anions such as chloride can affect dye uptake [32,33]. Other possible roles for ions is a modulation of the agonist binding site resulting in decreased agonist affinity for the P2X7 receptor [7] and a repression in the conformational changes that permit assembly or activation of the pore [34].

The controlled ionization marine solution we tested is a controlled ionization sea solution containing Ca^2+^, Mg^2+^, and Zn^2+^. It inhibited basal activation of P2X7 receptor in each tested cell line. Solutions rich in divalent cations could be easily used in therapeutics to help in inhibiting a new class of P2X7 receptor modulators.

These observations reveal a novel pathway for ocular toxicity in the ocular surface as well as in the retina. The implication of P2X7 receptor in different ocular stresses (i.e., preservatives and oxidative stress) confirms its pleiotropic activities.
